# Impact of Remote and Virtual Care Models on the Sustainability of Small Health Care Businesses: Perceptual Analysis of Small Clinics, Physician Offices, and Pharmacies in Colorado

**DOI:** 10.2196/23658

**Published:** 2021-02-25

**Authors:** Madhavan Parthasarathy, Jiban Khuntia, Rulon Stacey

**Affiliations:** 1 University of Colorado-Denver Denver, CO United States

**Keywords:** clinics, Colorado, COVID-19, electronic physician visits, entrepreneur, pharmacies, physician offices, small business, telehealth, telemedicine

## Abstract

**Background:**

Lockdowns and shelter-in-place orders during COVID-19 have accelerated the adoption of remote and virtual care (RVC) models, potentially including telehealth, telemedicine, and internet-based electronic physician visits (e-visits) for remote consultation, diagnosis, and care, deterring small health care businesses including clinics, physician offices, and pharmacies from aligning resources and operations to new RVC realities. Current perceptions of small health care businesses toward remote care, particularly perceptions of whether RVC adoption will synergistically improve business sustainability, would highlight the pros and cons of rapidly adopting RVC technology among policy makers.

**Objective:**

This study aimed to assess the perceptions of small health care businesses regarding the impact of RVC on their business sustainability during COVID-19, gauge their perceptions of their current levels of adoption of and satisfaction with RVC models and analyze how well that aligns with their perceptions of the current business scenario (SCBS), and determine whether these perceptions influence their view of their midterm sustainability (SUST).

**Methods:**

We randomly sampled small clinics, physician offices, and pharmacies across Colorado and sought assistance from a consulting firm to collect survey data in July 2020. Focal estimated study effects were compared across the three groups of small businesses to draw several insights.

**Results:**

In total, 270 respondents, including 82 clinics, 99 small physician offices, and 89 pharmacies, across Colorado were included. SRVC and SCBS had direct, significant, and positive effects on SUST. However, we investigated the effect of the interaction between SRVC and SCBS to determine whether RVC adoption aligns with their perceptions of the current business scenario and whether this interaction impacts their perception of business sustainability. Effects differed among the three groups. The interaction term SRVC×SCBS was significant and positive for clinics (*P*=.02), significant and negative for physician offices (*P*=.05), and not significant for pharmacies (*P*=.76). These variations indicate that while clinics positively perceived RVC alignment with the current business scenario, the opposite held true for small physician offices.

**Conclusions:**

As COVID-19 continues to spread worldwide and RVC adoption progresses rapidly, it is critical to understand the impact of RVC on small health care businesses and their perceptions of long-term survival. Small physician practices cannot harness RVC developments and, in contrast with clinics, consider it incompatible with business survival during and after COVID-19. If small health care firms cannot compete with RVC (or synergistically integrate RVC platforms into their current business practices) and eventually become nonoperational, the resulting damage to traditional health care services may be severe, particularly for critical care delivery and other important services that RVC cannot effectively replace. Our results have implications for public policy decisions such as incentive-aligned models, policy-initiated incentives, and payer-based strategies for improved alignment between RVC and existing models.

## Introduction

This study assessed the perceptions of small health care business owners toward remote and virtual care (RVC) services, which include telemedicine, telehealth, electronic physician visits, and internet-mediated communications and have become increasingly prevalent during COVID-19 with lockdowns, shelter-in-place orders, and other social distancing measures [1-4], effectively restricting patient access to in-person care. Consequently, in-person care–based small businesses have been severely impacted.

Hospitals and health systems are currently attempting to modify their care delivery methods in response to COVID-19. They have used various measures, such as discontinuing all elective procedures, converting ambulatory surgery suites to intensive care units, and moving a large portion of their supply offline to generate capacity in the event that the volume of patients with COVID-19 exceeds the projected volume. However, while hospitals and health systems have received financial incentives from the Federal Emergency Management Agency during COVID-19, no such incentives have been provided to the more than half a million small health care businesses, clinics, physician offices, blood banks, laboratories, and pharmacies, which form the traditional core of the health care ecosystem in the United States [5,6]. Consequently, many of these traditional small businesses have been experiencing considerable financial distress.

Over the past two decades, the health care industry had experienced a significant influx of entrepreneurs intending to capitalize on the country’s expanding health care expenditures, which amount for almost 20% of the gross domestic product, with an average of 5.5% growth projected through 2026 [7]. These entrepreneurs have often found themselves in a position of “doing good” as they provided much-needed services for the aging, ill, and disabled population at convenient locations. With the recent increase in the need for drug and substance abuse services and recent advancements in medical and technological treatments, they have entered a valuable niche in the health care market. Considering this trend in the health care industry, a plethora of businesses have been operating in the United States under the umbrella term of “clinics” to cater to the differing needs of care of the population. These clinics have been providing affordable care in areas such as physical and occupational therapy, diabetes care, kidney care, drug treatment, and rehabilitation centers, alternate medicine, hearing aid clinics, imaging centers, and nutrition or dietician services [8,9].

Similarly, small physician offices (ie, those with <5 physicians) constituted a significant component of the US health care system, garnering 54.5% of the total of 883.7 million visits to physician offices in 2016 [10]. Press reports reveal that small physician offices are under distress during COVID-19, and that “that won’t pay the light bill or the rent” [11], even though they note that these businesses are ready to respond to the challenge and provide care during the pandemic if they can receive incentives to stay operational.

Independent pharmacies in the United States have also been recently struggling, with their market share declining by approximately 10% in the last decade, although they still account for 35% of all retail pharmacies and a US $77.6 billion market share in 2017 [12]. Recent efforts to enhance price transparency have alleviated the “gag clauses,” which have served as a primary contributor to high out-of-pocket expenditures for drugs for patients [13,14] and have also resulted in a commensurate reduction in profit margins for some of those pharmacies. Stricter regulatory control has decreased the availability of over-the-counter medication now rather than a decade ago. Furthermore, physicians affiliated with health systems often electronically prescribe medication to be obtained from large pharmacy chains affiliated with the health care system, which have proliferated wider than before and are reportedly diverting large chunks of revenue from small, independently owned pharmacies [15].

Considering the lockdowns and shelter-in-place directives during the pandemic, consumers not only have greater access to web-based physician consultations but also have patronized online pharmacies [16]. Consequently, traditional pharmacies are losing their market share, leading to severe financial strain on them [17]. Indeed, more than half of the independent pharmacy owners are experiencing a negative revenue influx [18], with fewer individuals visiting stores [19]. Unlike large pharmaceutical companies, small pharmacies have also struggled to embrace the emerging RVC models and be sustainable, and this remains a concern [20].

This leads to the question of why these small businesses do not adopt RVC. We offer several conjectures to answer this question. First, RVC might be perceived as a temporary phenomenon, which could decline post COVID-19 because an online consultation cannot effectively replace an in-person consultation in many circumstances. Second, there may be a genuine fear that resource limitations may inhibit the ability of these small businesses to adopt RVC, and this may deter their sustained survival. Costs associated with digital infrastructure investment, platforms, connections, training, test runs, and scheduling services may be perceived as prohibitive. Third, whether organizations and insurance companies can reimburse RVC models remains a concern. Finally, assessment of the costs and benefits of adopting an RVC model can be difficult. The potentially expensive and arduous process of adopting an RVC model remains a major task, and there is no clarity on whether all patients and organizations would eventually transition to these models. Maintaining a brick-and-mortar store along with the RVC may be costly and overwhelming to these small business entities.

Small businesses can certainly benefit from integrating RVC processes into their existing business operations. Consumers, both on the demand and supply sides of care, are familiar with the use of cell phones, video chat platforms, text messaging, and cloud-based communication; hence, they can adopt a digital care platform without adequate training. Further, RVC models help overcome physical barriers, thereby increasing the reach of small businesses (and competition) to a much larger geographic area. Data collected by RVC systems may help identify how small firms may gain more business, connect patients with specialists in just-in-time mode, and improve branding.

This study aims to address these issues through a perceptual survey of small physician-owned businesses, clinics, and pharmacies. We sought to determine whether the owners of these businesses view RVC as a threat, whether they consider themselves capable of integrating the many opportunities accompanying RVC with their existing business model, and whether they fear midterm survival in the wake of RVC.

To answer these questions, we propose three conjectures based on our expectation that clinics, as opposed to small physician-owned businesses and pharmacies, will be better able to realize and leverage the value proposition of RVC because many clinics are affiliated with larger organizations that already have established digital platforms. Some of these may be retail or franchise establishments, perhaps connected to a larger health care system or hospital, which renders them more likely to be able to leverage the organization’s existing RVC or other digital platforms to their benefit to a larger extent than independent physician-owned businesses or pharmacies. Our conjectures are the following:

1. Small clinic owners will perceive value in aligning opportunities accompanying RVC models with their current business practices.
2. Small physician offices will not perceive value in aligning opportunities accompanying RVC models with their current business practices.
3. Small and independent pharmacy owners will not perceive value in aligning opportunities accompanying RVC models with their current business practices.

## Methods

### Recruitment

We conducted a focus group study with 8 health care experts via Zoom; they comprised 4 entrepreneurs familiar with the health care environment in Colorado, 2 physicians, and 2 executive health faculty members. The discussion centered around the impact of RVC on the sustainability of small physician offices, clinics, and pharmacies during COVID-19. The experts generally supported the notion that RVC adoption is posing a challenge to small health care businesses. As conjectured, the experts believed that clinics, by their scale and scope, would likely not only benefit from but also be able to rapidly integrate RVC into their business practices, more so than physicians and pharmacies. Furthermore, the experts believed that physician offices are most likely to directly compete with, and hence be threatened by, RVC. Clinics often provide in-person consultations; therefore, the focus group speculated that RVC was not a major direct threat to their sustainability as it was for physician offices. Furthermore, the experts speculated that small local pharmacies would be negatively impacted during COVID-19 and that consumers would increasingly tend to visit online pharmacies, thereby avoiding waiting in line at physical locations that may increase the risk of infection. To investigate whether these opinions were reflective of health care businesses in Colorado, our study was aimed at empirically validating these presumptions, using a scientifically collected sample of small health care businesses in Colorado.

### Data Collection

Considering the time sensitivity of this study, we solicited a professional consulting firm for data collection. The consulting firm sampled 445 small health care businesses in Colorado (135 clinics, 141 physician offices, and 169 pharmacies) through an opt-in approach during June-July 2020. Of these, 282 firms responded to the survey. In total, 12 observations were excluded owing to missing responses, resulting in a final sample size 270 businesses consisting of 82 clinics, 99 physician offices, and 89 pharmacies. The identities of the respondents were not disclosed.

The survey instrument used to collect the data is presented in Table 1. Questions regarding the current sustainability of the business in the subsequent 1-3 years were asked to the survey participants. Furthermore, satisfaction with the current state of the business and current remote care practices, if any, adopted by the business were measured. The survey was brief and succinct, taking only 5 minutes to complete, addressing only critical research questions and eliciting realistic responses.

The survey instrument, consisting of only 11 questions, was piloted with a sample of 22 respondents, leading to minor adjustments to a few items. Responses were coded, validated, and analyzed using STATA (version 14.2, StataCorp).

**Table 1:** Survey questionnaire and coding scheme.

Variable			Description		Questionnaire items		References	
**Dependent variables**								
		Sustainability of the physician office, clinic, or pharmacy (SUST)		Business owner’s perception of practice or business survival in the near future.		At an overall level, to what extent do you feel your clinic/practice/pharmacy is sustainable, considering the current COVID-19 scenario, in a span of the next 1-3 years?		Scale: 1 (least confident) to 5 (very confident)
**Independent variables**								
		Satisfaction with the current business scenario (SBCS)		Business owner’s satisfaction with the current practice and/or business scenario during COVID-19.		Questions: to what extent are you currently [during COVID-19 situation] satisfied with: (1) the overall clinic/practice/pharmacy relevant business scenario, situated across your city/town/county, (2) the current method of practice or business operations, and (3) the quality of day-to-day business transactions?		Scale: 1 (very dissatisfied) to 5 (very satisfied); Cronbach *α*=.79
		Satisfaction with remote and virtual care models (including telemedicine, telehealth, and e-visits) (SRVC)		Business owner’s satisfaction with the current (during COVID-19) remote care (telemedicine, telehealth, remote consulting, and e-visit provisions) provided by your clinic/practice/pharmacy.		Questions: to what extent are you currently (during COVID-19 situation) satisfied with the provisions and operations in your organization relevant to: (1) remote care, (2) telehealth and telemedicine, and (3) remote consulting and e-visits?		Scale: 1 (very dissatisfied) to 5 (very satisfied); Cronbach *α*=.82
**Control variables**								
	Age (AGE)		Age of the owner		My age is_________.		N/A^a^	
	Gender (GEN)		Gender of the owner		I am: (a) male and (b) female		N/A	
	Income (INC)		Net income of the owner from the business		My annual income from the clinic/practice/pharmacy relevant business: (1) less than US $50,000, (2) US $50,000-US $100,000, (3) US $100,000-US $200,000, (4) US $200,000-US $300,000, (5) US $300,000-US $500,000, (6) US $500,000-US $750,000, (7) US $750,000-US $1,000,000, and (8) higher than US $1,000,000.		N/A	
	Education (EDU)		Education of the owner		My education is: 1=illiterate, 2=up to middle school, 3=up to high school, 5=some college, 6=undergraduate level, and 7=graduate and above.		N/A	

^a^N/A: not applicable.

### Sample Descriptors and Demographics

Table 2 shows the descriptive statistics and pairwise correlations among the key variables. Most of the owners (mean 3.09, SD 0.6) believe that their business is on the verge of being sustainable, notwithstanding certain concerns. Owner satisfaction with the current business scenario was moderate to high (mean 3.16, SD 0.67). In contrast, owner satisfaction with remote care was low (mean 2.87, SD 0.76), probably owing to the unpreparedness and uncertainties associated with the delivery and rendering of remote care channels and options.

Of the 270 respondent business owners and chief executives, 229 (85%) identified as male. Respondent ages varied from 25-60 years, with 122 (45%) participants aged 25-35 years, 31 (12%) participants aged 35-45 years, 115 (43%) participants aged 45-55 years, and only 2 (0.01%) participants aged 55-60 years.

Further, 26 (10%) participants reported that they earned less than US $50,000 annually, 34 (13%) earned between US $50,000-US $100,000, 90 (33%) earned between US $100,000-US $200,000, 16 (6%) earned between US $300,000-US $500,000, 13 (5%) earned between US $500,000-US $750,000, 10 (4%) earned between US $750,000-US $1,000,000, and 19 (7%) earned more than US $1,000,000.

In addition, 1 (0.4%) participant reported having no education, 1 (0.4%) participant had 4 years of schooling, 4 (1.5%) participants had middle school education, 18 (7%) participants had high school education, 34 (13%) participants had some college education, 112 (42%) participants had an undergraduate degree, and 100 (37%) participants had graduate-level or higher education.

**Table 2:** Summary statistics and pairwise correlations among variables (N=270).

#	Variable	Mean (SD)	Minimum	Maximum	1	2	3	4	5	6	7
1	SUST^a^	3.09 (0.60)	1	4	1.00						
2	SCBS^b^	3.16 (0.67)	1	4	0.52	1.00					
3	SRVC^c^	2.87 (0.76)	1	4	0.48	0.31	1.00				
4	Age	37.45 (8.37)	25	60	0.02	0.07	0.06	1.00			
5	Gender	0.85 (0.36)	0	1	0.08	0.03	0.05	0.03	1.00		
6	INC^d^	4.67 (1.82)	2	8	–0.13	–0.14	–0.03	0.03	0.00	1.00	
7	EDU^e^	6.03 (1.02)	1	7	–0.14	–0.10	–0.05	–0.14	0.07	0.17	1.00

^a^SUST: sustainability of the physician office, clinic, or pharmacy.

^b^SCBS: satisfaction with the current business scenario.

^c^SRVC: satisfaction with remote and virtual care models (including telemedicine telehealth and e-visits).

^d^INC: income.

^e^EDU: education.

### Study Variables

We used a dependent variable reflecting the owner’s perception of the business sustainability of physician offices, clinics, or pharmacies in the near future (SUST). This variable was measured using a single-item question. On average, SUST had a mean score of 3.09 (SD 0.6) of 5, indicating that most businesses are somewhat confident in remaining sustainable (Table 2).

Two independent variables were of interest in this study. First, the independent variable of satisfaction with the current business scenario (SCBS) measured the owner’s satisfaction with the current practice or the business scenario during COVID-19. This variable was operationalized with three questions on the business scenario across the city/town/county, the current ways of practice or business operations, and the quality of day-to-day business transactions during the pandemic. The internal consistency of the items was high, as reflected by their Cronbach *α* of .79 and a variable response of 3.16 (SD 0.67) (Table 2).

Second, the satisfaction with RVC (SRVC) models indicated the owner’s or chief executive’s satisfaction with current remote care (telemedicine, telehealth, remote consulting, and e-visit provisions) provided by their clinics, practice offices, or pharmacies. This variable was operationalized with using three questions on the provisions and aligned operations in the clinic, physician office, or pharmacy relevant to remote care, telehealth, and telemedicine, and remote consulting during COVID-19 (Table 1). The items had high internal consistency with a Cronbach *α* of .82 and a variable response of 2.87 (SD 0.76) (Table 2).

We were particularly interested in exploring how owners perceived the importance of remote care to remain competitive and mitigate concerns relevant to their current business scenario during COVID-19. To explore this, we established an interaction variable multiplying SCBS and SRVC (SCBS×SRVC), which was used in the models to reflect the net effect of satisfaction with the current business and existing remote care provisions on perceptions of business sustainability.

In addition to these key variables of interest, a limited set of control variables such as age, gender, income, and education were included to account for potential counterfactual explanations relevant to our models.

### Econometric Analysis

The empirical model used herein analyzed the impact of owners’ perceptions of their current business scenario and their ensuing use of remote care systems on the perceived sustainability of their small businesses. We used an ordinary least square estimation as follows:

Y[SUST]_i_ = β_0_ + β_1_ SCBS_i_ + β_2_ SRVC_i_ + β_3_ SCBS_i_ × SRVC_i_ + Controls_i_ + ε_I_ (1)

where *Y* is the dependent variable; *β_0_*, *β_1_*, *β_2_*, and *β_3_* are the parameter coefficients; and ε is the disturbance associated with each observation. We estimated this model for all businesses in our sample and then separately for the subsamples of clinics, physician offices, and pharmacies.

## Results

Table 3 presents the estimation results obtained using equation (1) and summarizes the estimates for the entire sample and individually for clinics, physician offices, and pharmacies.

We found that both SCBS and SRVC variables have significant, positive direct effects on SUST, which indicates that the effort invested in the current situation and in adopting remote care systems influenced the sustainability of the businesses as perceived by the owners. However, this study aimed to investigate whether remote care provision was perceived as adequate in the current business scenarios to influence business sustainability. Accordingly, the coefficients of the interaction variable SCBS×SRVC were further analyzed.

**Table 3:** Estimation results (N=270).

Variables	Clinics		Physician offices			Pharmacies				Full sample	
	SUST^a^	*P* value		SUST	*P* value		SUST	*P* value	SUST		*P* value
SCBS^b^, mean (SE)	0.556 (0.094)	<.001		0.187 (0.097)	.06		0.394 (0.101)	<.001	0.352 (0.056)		<.001
SRVC^c^, mean (SE)	0.235 (0.070)	.001		0.159 (0.076)	.04		0.351 (0.070)	<.001	0.277 (0.041)		<.001
SCBS×SRVC, mean (SE)	0.274 (0.110)	.02		-0.182 (0.092)	.05		0.026 (0.083)	.76	–0.011 (0.052)		.83
Age, mean (SE)	0.000 (0.006)	.95		–0.000 (0.007)	.94		–0.001 (0.006)	.81	–0.003 (0.004)		.46
Gender, mean (SE)	–0.028 (0.175)	.87		0.141 (0.137)	.31		0.177 (0.133)	.19	0.091 (0.081)		.26
Income, mean (SE)	–0.027 (0.092)	.77		–0.029 (0.024)	.23		–0.004 (0.026)	.87	–0.017 (0.016)		.31
Education, mean (SE)	–0.059 (0.053)	.27		–0.037 (0.051)	.47		–0.100 (0.050)	.05	–0.048 (0.029)		.10
Constant, mean (SE)	3.721 (0.569)	<.001		3.331 (0.461)	<.001		3.915 (0.366)	<.001	3.626 (0.243)		<.001
Observations, n	82	N/A^d^		99	N/A		89	N/A	270		N/A
*R^2^*	0.477	N/A		0.329	N/A		0.492	N/A	0.393		N/A
Adjusted *R*^2^	0.427	N/A		0.278	N/A		0.448	N/A	0.377		N/A
*F* test (*df*)	9.638 (7)	N/A		6.383 (7)	N/A		11.200 (7)	N/A	24.280 (7)		N/A
Prob>F	0.000	N/A		0.000	N/A		0.000	N/A	0.000		N/A

^a^SUST: sustainability of the physician office, clinic, or pharmacy.

^b^SCBS: satisfaction with the current business scenario.

^c^SRVC: satisfaction with remote and virtual care models (including telemedicine telehealth and e-visits).

^d^N/A: not applicable.

First, we found that for clinics, the estimation coefficient was positive and significant (*β*=.274; *P*=.02). This finding indicated a positive interaction between the current business scenario and the adoption of remote care platforms, reflecting higher perceived business sustainability. Second, for physician offices, the opposite trend was observed, in that the estimation coefficient was negative and significant (*β*=–.182; *P*=.05)*.* This finding indicates a negative interaction between their satisfaction with RVC and their satisfaction with their current business scenario, reflecting lower future sustainability of their businesses. Third, for pharmacies, the estimation coefficient was not significant (*β*=.026; *P*=.76)*.* This finding indicates that pharmacy owners did not perceive the interaction between their satisfaction of remote care platforms and the current business scenario to be associated with the sustainability of their businesses. Finally, the estimation coefficient of SCBS×SRVC of the complete sample was not significant (*P*=.83), probably owing to an aggregation of positive and negative interactions across the subsamples.

## Discussion

### Practice and Policy Implications of the Findings

RVC adoption (ie, telemedicine, telehealth, e-visits, and similar internet-enabled treatment options) has grown significantly during COVID-19. RVC fulfills a widespread patient-centric need for access to services of the US health care sector, which is likely to be a high-growth sector in the near future. The growth of RVC has impacted traditional small health care businesses that have traditionally relied on in-person care and serve a local geographic area. Our study was aimed at measuring the perceptions of RVC of these small business owners and determining whether they believe that RVC would threaten their survival or sustainability or, in contrast, if they perceived RVC as a growth opportunity.

Our study assessed the perceptions of three categories of health care providers: physician offices, clinics, and pharmacies. We found that clinic owners were most likely to believe that their adoption of the RVC model interacted positively with their perceptions of the current business scenario, which, in turn, led to a more positive perception of their business sustainability over the subsequent 1-3 years. In other words, their satisfaction with the RVC model was synergistic with their satisfaction with current business practices, and this was associated with a more optimistic opinion of the firm’s midterm business survival. These findings may be certainly moderated by the type of clinic in question. A majority of clinics, such as physical therapy businesses, imaging services, or diabetes care centers that depend on in-person visits, may not perceive RVC as a direct threat. For them, adoption of a digital platform, particularly to enhance efficiency, customer service, and information provision, may complement their services and extend their reach to a younger, more digitally savvy audience.

However, this was not the case with physician offices. The interaction between their satisfaction with the current business scenario and RVC was negative, resulting in a lower perception of their firm’s midterm sustainability. In other words, these business owners speculated that RVC, when combined with the existing business scenario, was negatively associated with their likely midterm survival. While the interaction between their perception of the current business scenario and RVC was negative for pharmacies as well, the coefficient was nonsignificant, suggesting that pharmacies did not consider their satisfaction with RVC and the current business scenario to impact their business’s midterm sustainability.

Overall, clinics perceived RVC and the current business scenario to be positively associated with business sustainability. In contrast, physician offices speculated that RVC adoption along with the current in-person business scenario was negatively associated with their business’s midterm survival.

Many of these small businesses do not qualify for financial assistance from the Federal Emergency Management Agency during COVID-19, and this has significantly, albeit temporarily, decelerated business operations during the pandemic and caused them financial distress. Furthermore, their inability to rapidly adopt a satisfactory and synergistic RVC model to atone for the loss of traditional customers further aggravated this problem. The recent policy-level incentive to render RVC models a permanent provision in the US health care system, without considering the opinions and realities of small health care businesses (which are the backbone of the traditional health care delivery system), seems myopic. RVC cannot replace traditional in-person health care provision in all circumstances, and if physician offices and other small health care businesses cannot survive the competition posed by the RVC model, the results could lead to a marked deficit in critical care. Hence, new technologies should be adopted in a nuanced manner, with policy-level support for these small businesses, thus laying the foundation for gradual and progressive RVC adoption [18].

Certain other issues also need careful policy restructuring: (1) linking insurance payments and payor guarantees to encourage small businesses to adopt the RVC model; (2) developing creative, new consumer-centric models in this context; (3) ensuring affordable RVC platforms for small businesses with reliable technological and infrastructural logistics to ensure that remote care is error-free and of the highest quality; and (4) integrating RVC models with traditional brick-and-mortar health care models that have been established in accordance with the high-touch concept. If remote RVC technology can complement and not compete with in-person treatment models, such a system would have a high combined efficiency. For instance, technologies such as remote patient monitoring and vital reading systems can help improve the impact of traditional systems.

A related implication is to prepare current and next-generation patients and care providers to embrace RVC-related changes that have not been and are not a part of the curriculum in medical or business schools. Medical practitioners may not be good business persons and vice versa. Small physician offices have been particularly faced with this conundrum while facing several regulatory and practice-level changes, which were not a part of their curriculum. Advancements in RVC involve artificial intelligence, machine learning, data science, and above all, a set of customer-oriented, internet-based patient communication and payment or profit models, which need both medical and business skills. The future curriculum should foster the acquisition of business knowledge and skills.

### Conclusions

The rapid spread of COVID-19 has yielded unprecedented challenges. Simultaneously, it has helped to rapidly accelerate the adoption of RVC models. RVC serves as a starting point for several innovations that would potentially modify the existing care models and, when used carefully, may lead to a synergistic mixed model of in-person and internet-based care provision. However, our results show that RVC adoption may have progressed too rapidly, with small businesses struggling to integrate RVC technologies with their existing in-person platforms. Physician offices, in particular, are experiencing incompatibility between their current business models and RVC technologies and are concerned about the potential impact of RVC adoption on their midterm survival. If a significant proportion of brick-and-mortar physician offices become nonoperational, the collective impact of such a closure on the US health care system could be catastrophic. Hence, careful consideration of the adoption of RVC models (potentially including business education, government incentives, and support for small businesses) is vital to the sustained adoption and functionality of RVC models. While it is clear that the future of health care depends on these transformative models, the complexity lies in managing these models with appropriate support, training, reimbursement, and incentive models for small businesses, while meeting the expectations and demands of patient-centric health care delivery.

## Abbreviations

RVC: remote and virtual care
SCBS: satisfaction with the current business scenario
SRVC: satisfaction with remote and virtual care models
SUST: sustainability of the physician office, clinic, or pharmacy
